# Status quo der betrieblichen Gesundheitsförderung in Inklusionsbetrieben. Potenziale und Herausforderungen für die Zukunft

**DOI:** 10.1007/s11553-022-01003-6

**Published:** 2022-12-01

**Authors:** Ilona Efimov, Ann-Christin Kordsmeyer, Volker Harth, Stefanie Mache

**Affiliations:** Zentralinstitut für Arbeitsmedizin und Maritime Medizin (ZfAM), Universitätsklinikum Hamburg-Eppendorf (UKE), Seewartenstr. 10 | Haus 1, 20459 Hamburg, Deutschland

**Keywords:** Arbeitsbedingungen, Integration, Arbeitsbezogene Gesundheit, Behinderungen, Psychische Gesundheit, Working conditions, Social integration, Occupational health, Disabled persons, Mental health

## Abstract

**Hintergrund:**

Inklusionsbetriebe beschäftigen nach §§ 215–218 SGB IX 30–50 % Menschen mit Behinderungen auf dem ersten Arbeitsmarkt und sind seit dem 01.01.2018 verpflichtet, Maßnahmen zur betrieblichen Gesundheitsförderung (BGF) anzubieten.

**Ziel der Arbeit:**

Der Artikel soll eine Übersicht zum aktuellen Stand der Forschung zur BGF in Inklusionsbetrieben bieten und Potenziale für die Praxis ableiten.

**Material und Methoden:**

Mithilfe einer explorativen Literaturrecherche werden bisher verfügbare Erkenntnisse zur Arbeits- und Gesundheitssituation von Menschen mit Behinderungen und Leitungskräften aus Inklusionsbetrieben sowie die Umsetzung, Herausforderungen und Bedarfe im Bereich der BGF zusammengefasst. Aus den Erkenntnissen werden Forschungslücken identifiziert und praktische Implikationen abgeleitet.

**Ergebnisse:**

Die Übersichtsarbeit zeigt auf, dass Inklusionsbetriebe für Beschäftigte mit Behinderungen eine Vielzahl an Ressourcen bereitstellen, um den Arbeitsanforderungen am ersten Arbeitsmarkt zu begegnen. Leitungskräfte hingegen erleben die Bewältigung von sozialen und wirtschaftlichen Anforderungen im Inklusionsbetrieb als Arbeitsanforderung ihrer Tätigkeit. Bisherige Forschungsergebnisse verdeutlichen, dass die Netzwerkbildung von kleinen und mittelständischen Unternehmen das Potenzial bietet, BGF in Inklusionsbetrieben zu fördern.

**Schlussfolgerung:**

Der Artikel elaboriert die Arbeits- und Gesundheitssituation von Beschäftigten und Leitungskräften in Inklusionsbetrieben sowie den Status quo von BGF-Maßnahmen in Inklusionsbetrieben. Der aktuelle Stand der Forschung verdeutlicht, dass noch ein großer Bedarf zur Implementierung und Evaluation von BGF-Maßnahmen in Inklusionsbetrieben besteht. Identifizierte Herausforderungen bei der Umsetzung von BGF in Inklusionsbetrieben sollten in der Praxis entsprechend adressiert werden.

## Abstract (en)

**Background:**

Social firms employ 30–50% people with disabilities on the general labour market according to §§ 215–218 SGB IX and are obliged to offer workplace health promotion (WHP) since 01 January 2018.

**Objectives:**

This article aims to provide an overview of the current state of research on WHP in social firms and to derive potentials for practice.

**Materials and methods:**

In an exploratory literature overview, previously available findings on the work and health situation of people with disabilities and supervisors from social firms as well as the implementation, challenges and needs for WHP are researched and summarized. Research gaps are identified and practical implications derived from the research findings.

**Results:**

This overview highlights that social firms provide a variety of resources for employees with disabilities to manage job demands on the primary labour market. Supervisors, on the other hand, perceive coping with social and economic demands in social firms as a job demand. Previous research indicates that the development of networks of small and medium-sized enterprises has the potential to promote inclusive WHP.

**Conclusion:**

This article elaborates the work and health situation of employees and leaders in social firms as well as the status quo of WHP measures in social firms. The empirical state of research illustrates the need for more research on the implementation and evaluation of WHP measures in social firms. In practice, there is also potential for improvement in addressing existing challenges to WHP in social firms.

## Einleitung

In Deutschland leben über 7,9 Mio. Menschen mit einer Schwerbehinderung. Eine Behinderung liegt vor, wenn über einen längeren Zeitraum körperliche, seelische, geistige oder Sinnesbeeinträchtigungen erlebt werden, die eine Teilhabe an der Gesellschaft erschweren. Der Grad der Behinderung (GdB) wird in Zehnerschritten (von 20–100) festgelegt, wonach eine Schwerbehinderung ab einem GdB von 50 festgelegt wird (§ 2 Neuntes Buch Sozialgesetzbuch [SGB IX]). Derzeit befinden sich ca. 40 % der schwerbehinderten Menschen in Deutschland im arbeitsfähigen Alter (zwischen 25 und 64 Jahren; [32]). Jedoch fällt der Anteil erwerbstätiger Menschen mit Schwerbehinderung in Deutschland (46,9 %) geringer aus im Vergleich zu Menschen ohne Behinderung (75,2 %). Auch wenn der Anteil erwerbstätiger schwerbehinderter Menschen kontinuierlich steigt, wurde dieser Wachstumstrend im Jahr 2020 durch die COVID-19-Pandemie („coronavirus disease 2019“) vorerst gestoppt. Die Arbeitslosigkeit schwerbehinderter Menschen wird stärker durch rechtliche Rahmenbedingungen und die demografische Entwicklung beeinflusst. Somit sind schwerbehinderte Arbeitslose im Durchschnitt älter und länger arbeitslos als nicht-schwerbehinderte Arbeitslose [4]. Erwerbsarbeit bietet für Menschen mit Behinderungen jedoch verschiedene positive Einflussmöglichkeiten, u. a. auf den Genesungsprozess von Menschen mit psychischen Erkrankungen, der Erlangung einer beruflichen Identität und Stärkung eines Gefühls der Selbstbestimmung [7]. Daher sollten Beschäftigungsverhältnisse für Menschen mit Behinderungen am ersten Arbeitsmarkt entsprechend gestärkt und gefördert werden.

Inklusionsbetriebe (oder -abteilungen) stellen in Deutschland eine besondere Unternehmensform dar, welche gemäß Artikel 27 der UN-Behindertenrechtskonvention Menschen mit Behinderungen eine gleichberechtigte Teilhabe am ersten Arbeitsmarkt ermöglichen sollen [5]. Im Vergleich zu anderen Unternehmen werden ca. 30–50 % Menschen mit psychischen oder geistigen, Sinnes‑, Körper- oder Mehrfachbehinderungen in Inklusionsbetrieben beschäftigt, welche sonst eine Benachteiligung auf dem allgemeinen Arbeitsmarkt erfahren (§ 215 SGB IX). In den Betrieben sollen schwerbehinderte Menschen auf den Übergang in einen Betrieb auf dem allgemeinen Arbeitsmarkt vorbereitet werden, beispielsweise nach Beendigung der schulischen Laufbahn, einer Werkstatt für behinderte Menschen (WfbM) oder einer Langzeitarbeitslosigkeit (§ 215 SGB IX). Neben der Gewährleistung unterstützender Arbeitsbedingungen ermöglichen Inklusionsbetriebe nach § 216 SGB IX u. a. eine arbeitsbegleitende Betreuung oder bedarfsorientierte Maßnahmen der beruflichen Weiterbildung. Inklusionsbetriebe können für den Ausbau, die Erweiterung, Modernisierung und Ausstattung ebenso wie für eine betriebswirtschaftliche Beratung und für besonderen Aufwand finanzielle Mittel aus der Ausgleichsabgabe erhalten (§ 217 SGB IX). Allgemein sind Arbeitgeber:innen in Deutschland gesetzlich verpflichtet, eine verschriebene Anzahl der Arbeitsplätze mit schwerbehinderten Menschen zu besetzen. Wird diese Quote nicht erfüllt, muss eine Ausgleichsabgabe gezahlt werden (§ 160 SGB IX).

Seit 2018 sind Inklusionsbetriebe im Gegensatz zu anderen Betrieben auf dem ersten Arbeitsmarkt dazu verpflichtet, über den allgemeinen Arbeits- und Gesundheitsschutz hinaus Maßnahmen der betrieblichen Gesundheitsförderung (BGF) anzubieten. BGF-Maßnahmen können sowohl darauf ausgerichtet sein, individuelles Verhalten (Verhaltensprävention) als auch Arbeitsbedingungen zu verändern (Verhältnisprävention; [12]). Nach dem aktuellen Stand der Forschung wird im Sinne eines ganzheitlichen Ansatzes die Kombination von sowohl verhaltens- als auch verhältnispräventiven BGF-Maßnahmen am wirkungsvollsten erachtet [13]. Zudem konnte ein gesundheitlicher wie auch ökonomischer Nutzen von BGF-Maßnahmen für Betriebe nachgewiesen werden [14, 20, 25, 29, 38]. Trotz des gesundheitsförderlichen Potenzials und der Gesetzesänderung zur verpflichtenden Umsetzung von BGF-Maßnahmen in Inklusionsbetrieben (§ 216 SGB IX) besteht bislang weiterhin ein großer Bedarf an angepassten BGF-Angeboten für Inklusionsbetriebe in der Praxis [31] sowie ein großer Forschungsbedarf zu BGF in Inklusionsbetrieben [19].

## Arbeitsbedingungen in Inklusionsbetrieben und Abgrenzung zu anderen Beschäftigungsmöglichkeiten

### Arbeitsbedingte Ressourcen und Arbeitsanforderungen von Beschäftigten mit Behinderungen

Nach dem aktuellen Stand der Forschung zeigt sich, dass in Inklusionsbetrieben eine spezifische Anforderungs-Ressourcen-Situation für Beschäftigte mit Behinderungen besteht. Vergleichbar mit deutschen Inklusionsbetrieben erweisen sich im internationalen Kontext sog. „social firms“, welche eine sinnvolle Beschäftigung für benachteiligte Personen auf dem ersten Arbeitsmarkt schaffen und eine signifikante Anzahl von Menschen mit Behinderungen (von mindestens 25 %) beschäftigen [8]. Auch in diesem Rahmen wird ein hohes Maß an Unterstützung für die Beschäftigten unter Wahrung von Autonomie, Selbstbestimmung und Partizipation am Arbeitsplatz beschrieben [8]. In den meisten Studien zur Arbeits- und Gesundheitssituation werden neben Menschen mit Lern‑, Sinnes- oder geistigen Behinderungen bisher überwiegend Menschen mit psychischen Erkrankungen befragt [19]. Im Bereich der arbeitsbedingten Ressourcen für Beschäftigte mit Behinderungen wird deutlich, dass auf der einen Seite eine Vielzahl solcher in den Bereichen Arbeitsinhalte, Arbeitsorganisation, soziale Beziehungen und der Arbeitsumgebung geboten wird [19, 22]. Auch aktuelle Ergebnisse aus dem Kontext deutscher Inklusionsbetriebe beschreiben, dass Beschäftigte über ein hohes Maß an erfüllenden und als sinnvoll erlebten Arbeitsaufgaben (z. B. im Kundenkontakt) verfügen [10]. Im Bereich der Arbeitsorganisation berichten Beschäftigte im Vergleich zu anderen Betrieben auf dem ersten Arbeitsmarkt eine Vielzahl an Ressourcen in Inklusionsbetrieben. So werden flexible Arbeitsbedingungen, ein an die individuellen Kapazitäten angepasstes Arbeitspensum, eine angemessene Verteilung der Arbeit innerhalb des Teams ebenso wie etablierte Arbeitsprozesse mit einer klaren Verteilung von Aufgaben und Verantwortung als unterstützend erlebt. Weiterhin werden eine regelmäßige Kommunikation im Team oder die Flexibilität in der Gestaltung von Pausen als Ressourcen wahrgenommen. Ein verlässlicher Lohn und Arbeitsplatzsicherheit in Inklusionsbetrieben stellen weitere charakteristische Ressourcen dar, etwa im Vergleich zu WfbM, in denen ein Arbeitsentgelt (§ 219 Abs. 1 SGB IX) gezahlt wird [10, 19]. Zudem können auch Weiterbildungs- und Partizipationsmöglichkeiten am Arbeitsplatz als Ressourcen im Vergleich zu anderen Betrieben auf dem ersten Arbeitsmarkt erlebt werden [19]. Die Ergebnisse statistischer Berechnungen verdeutlichen zudem, dass eine hohe zeitliche Flexibilität und Weiterbildungsmöglichkeiten zusätzlich die Beschäftigungsdauer in Inklusionsbetrieben erhöhen können [35]. Im Bereich der sozialen Beziehungen werden sowohl im internationalen Forschungsstand als auch in deutschen Inklusionsbetrieben insbesondere ein hoher Zusammenhalt im Team und eine harmonische Arbeitsatmosphäre, welche durch einen wertschätzenden, nicht-diskriminierenden Umgang, Geduld im Umgang mit Fehlern sowie gegenseitige Akzeptanz von Schwächen von Beschäftigten mit Behinderungen, berichtet. Neben der sozialen Unterstützung durch das Team können auch Leitungskräfte eine wichtige Ressource am Arbeitsplatz darstellen. Bei der Konfliktlösung oder auch bei wertschätzendem, respektvollem Feedback und der Kommunikation von Anerkennung werden Leitungskräfte unterstützend erlebt [10, 19]. Ergänzend können auch weitere Akteure wie private Beziehungen als Ressource zur Bewältigung der Arbeitsaufgaben fungieren [19]. Die Ergebnisse internationaler Studien verdeutlichen hierbei, dass sich eine hohe soziale Unterstützung am Arbeitsplatz positiv auf die Arbeitszufriedenheit, die Produktivität, die Motivation und das Arbeitsengagement der Beschäftigten auswirken kann [9, 33, 34, 36, 37]. Im Bereich der Arbeitsumgebung werden für deutsche Inklusionsbetriebe besonders die Berücksichtigung von Arbeitssicherheit sowie die Bereitstellung von Snacks und Getränken in Meetings als Ressourcen am Arbeitsplatz hervorgehoben [10]. Andere Studien berichten, dass Beschäftigte die Bereitstellung von Arbeitsmitteln sowie die Möglichkeit, die Arbeitsumgebung zu verändern (z. B. den Lärmpegel) oder einen Sitzplatz zu haben, positiv bewerten [8, 21]. Des Weiteren werden in der Literatur persönliche Ressourcen wie eine arbeitsbezogene Selbstwirksamkeit oder Selbstbewusstsein unterstützend bei der Bewältigung der Arbeitsaufgaben von Beschäftigten mit Behinderungen erlebt [19].

Auf der anderen Seite zeigen internationale wie auch nationale Forschungsergebnisse, dass derzeit eine geringe Studienlage zu den Arbeitsanforderungen von Beschäftigten mit Behinderungen in Inklusionsbetrieben besteht [10, 19]. Im Bereich der Arbeitsinhalte werden Beschäftigte mit Behinderungen mit Arbeitsaufgaben konfrontiert, die sie beispielsweise durch vermehrte Einzelarbeit oder sich wiederholenden Aufgaben als beanspruchend erleben [10]. Im Bereich der Arbeitsorganisation nehmen Beschäftigte, ähnlich wie in anderen Betrieben auf dem ersten Arbeitsmarkt, teilweise eine hohe Arbeitsmenge bzw. Zeitdruck, Herausforderungen in der Zusammenarbeit wie eine ungerechte Verteilung von Arbeit im Team, frühe Arbeitszeiten oder eine unzureichend erlebte Entlohnung belastend wahr [10, 19]. Weitere Einschränkungen in Betrieben, z. B. durch unvollständige oder schlechte Informationsweitergabe oder Arbeitsunterbrechungen können zudem einen negativen Einfluss auf die Arbeitsleistung von Beschäftigten, ihre Arbeitszufriedenheit und ihre Motivation haben, den Job zu behalten [9, 36, 37]. Im Bereich der sozialen Beziehungen werden sich in Konflikte einmischende Teammitglieder oder auch der Druck der Leitungskraft, welcher sich entsprechend auf die Beschäftigten überträgt, als Belastungen am Arbeitsplatz berichtet [10, 19]. Ähnlich wie in anderen Betrieben am ersten Arbeitsmarkt können im Bereich der Arbeitsumgebung je nach Branche ebenfalls eine hohe körperliche Anstrengung, hohe Arbeitstemperaturen oder Lärm am Arbeitsplatz beanspruchend erlebt werden [10, 19]. Zusätzlich können persönliche Faktoren wie die Symptomschwere der jeweiligen Behinderung oder Selbststigmatisierung die Belastungssituation am Arbeitsplatz negativ beeinflussen [19]. Während der COVID-19-Pandemie werden zudem weitere Herausforderungen für Beschäftigte mit Behinderungen in Inklusionsbetrieben sichtbar: u. a. der plötzliche Wegfall von Arbeit, Strukturen und sozialen Kontakten, Ängste vor dem Jobverlust, finanzielle Herausforderungen, ein Mangel an Bewegung – sowie eine branchenspezifische Mehrarbeit aufgrund erhöhter Auftragslage [17].

### Arbeitsbedingte Ressourcen und Arbeitsanforderungen von Leitungskräften

Auch für die Leitungskräfte in Inklusionsbetrieben zeigt sich, dass unterschiedliche Ressourcen in den Bereichen Arbeitsinhalte, Arbeitsorganisation, soziale Beziehungen und der Arbeitsumgebung wahrgenommen werden [16]. Spezifisch für Inklusionsbetriebe werden die Arbeitsinhalte für Leitungskräfte durch eine von ihnen hohe wahrgenommene Bedeutung der Arbeit charakterisiert (z. B. wenn die Beschäftigten an Stabilität gewinnen, ihre Arbeitszeit erhöhen oder neue Kompetenzen erlernen). Ebenso werden die Arbeitsaufgaben als abwechslungsreich beschrieben, welche sich aus fachlichen und pädagogischen Anteilen zusammensetzen [16]. Diese Ergebnisse werden von einer weiteren Online-Befragung mit Leitungskräften aus deutschlandweiten Inklusionsbetrieben bestätigt, wonach ihre wahrgenommene Bedeutung der Arbeit einen Einfluss auf das Arbeitsengagement der Leitungskräfte hat [15]. Weitere Ressourcen, die auch für andere Leitungskräfte auf dem ersten Arbeitsmarkt bekannt sind [41], werden etwa in Form von Handlungsspielräumen ergänzt [16]. Als charakteristisch für den Bereich der Inklusionsbetriebe erweist sich zudem, dass teilweise bereits Schulungen und Seminare angeboten werden, z. B. zum Umgang mit Menschen mit psychischen Erkrankungen [8, 16]. Weiterhin können einige Leitungskräfte von den Arbeitszeiten profitieren, zu denen überwiegend keine Abend- oder Wochenendarbeit gehört [16]. Auch aus anderen Betrieben bekannte Ressourcen bestehen durch Mitgestaltungsmöglichkeiten hinsichtlich der Strukturen, regelmäßige Treffen bzw. Austausch im Betrieb, feste Vertretungsregelungen oder eine Zusammenarbeit und Vernetzung mit externen Organisationen. Die sozialen Beziehungen der Leitungskräfte sind überwiegend von einer positiven Arbeitsatmosphäre gekennzeichnet [16]. In diesem Zusammenhang zeigt sich zusätzlich die soziale Unterstützung durch pädagogisch geschulte Mitarbeiter:innen, die neben der Unterstützung der Teammitglieder oder der Geschäftsführung eine zentrale Rolle einnehmen. Auch die Ergebnisse der Online-Befragung von Leitungskräften aus Inklusionsbetrieben zeigen, dass eine wahrgenommene organisationsbezogene Unterstützung im Betrieb einen Einfluss auf Burnout-Symptome und das Arbeitsengagement der Leitungskräfte hat [15]. Weiterhin beschreiben einige Leitungskräfte Wertschätzung, Kundenzufriedenheit oder die Bereitstellung eines sozialpsychiatrischen Dienstes als Ressourcen. Im Bereich der Arbeitsumgebung unterscheiden sich Inklusionsbetriebe weniger von anderen Betrieben auf dem ersten Arbeitsmarkt und nennen ein ergonomisches Arbeitsumfeld, eine geräumige Arbeitsumgebung oder die Nutzung von Arbeitsmitteln als Ressourcen [16].

Auf der anderen Seite erleben Leitungskräfte in Inklusionsbetrieben verschiedene Arbeitsanforderungen in den genannten Bereichen [16]. Charakteristisch für Inklusionsbetriebe werden für Leitungskräfte Herausforderungen in der Anleitung von Tätigkeiten beschrieben, beispielsweise wenn Beschäftigte mit Behinderungen weniger vorausschauend planen können. Emotionale Anforderungen stellen einen weitere Arbeitsanforderung dar, etwa wenn private Ängste und Sorgen der Beschäftigten an die Leitungskräfte herangetragen werden [16]. Andere Arbeitsanforderungen, die auch aus anderen Bereichen bekannt sind [30], entstehen durch Interaktionen wie etwa im Kontakt mit Kundschaft [16]. Charakteristische arbeitsorganisatorische Arbeitsanforderungen werden zudem durch kollidierende wirtschaftliche und soziale Anforderungen genannt [16, 24], z. B. wenn Leitungskräfte bei hoher Arbeitsbelastung mit akuten Herausforderungen von Beschäftigten umgehen müssen. Damit eng in Verbindung stehen kurzfristige Ausfälle von Beschäftigten mit psychischen Erkrankungen, welche von der Leitungskraft im Inklusionsbetrieb entsprechend abgefangen bzw. kompensiert werden müssen [16]. Weitere Herausforderungen entstehen zudem in der Zusammenarbeit mit den Beschäftigten (z. B. aufgrund von Vergesslichkeit der Beschäftigten oder wenn häufig die gleichen Fragen gestellt werden). Ebenso wird teilweise von Verhandlungen mit dem Integrationsamt berichtet [16]. Auch die Online-Befragung der Leitungskräfte kann diese Tendenzen entsprechend untermauern. Quantitative Arbeitsanforderungen können demnach einen Einfluss auf Burnout-Symptome der Leitungskräfte in Inklusionsbetrieben haben [15]. Diese Anforderungen werden jedoch auch in anderen Betrieben auf dem ersten Arbeitsmarkt berichtet, u. a. aufgrund von Zeitdruck und einer hohen Arbeitsintensität [16, 41]. Weiterhin werden Überstunden, undefinierte Kommunikationsstrukturen, eine ständige Erreichbarkeit, eine geringe finanzielle Entlohnung, ein langer Arbeitsweg oder eine fehlende Einarbeitung beschrieben [16]. Auch im Bereich der sozialen Beziehungen zeigen sich verschiedene Inklusionsbetriebs-spezifische Arbeitsanforderungen. So wird eine fehlende soziale Unterstützung deutlich, wenn keine weitere Person ohne Behinderung in der Abteilung angestellt ist. Zudem zählt das Konfliktmanagement zu den Aufgaben einer Leitungskraft, z. B. bei Konflikten aufgrund unterschiedlicher Fähigkeiten der Beschäftigten. Weitere charakteristische Arbeitsanforderungen bestehen durch Herausforderungen in der Kommunikation mit gehörlosen Beschäftigten. Weniger spezifische Arbeitsanforderungen wie eine geringe Wertschätzung werden ebenfalls genannt. Auch die Arbeitsanforderungen in der Arbeitsumgebung wie Hitze, begrenzte Räumlichkeiten, schweres Heben oder ständiges Stehen sind bereits aus anderen Betrieben des ersten Arbeitsmarktes bekannt [16].

Auch für die Leitungskräfte in Inklusionsbetrieben werden verschiedene zusätzliche Anforderungen während der COVID-19-Pandemie sichtbar: Die Leitungskräfte müssen mit weniger Personal arbeiten, Aufgaben entsprechend abfangen, Konflikte in der Zusammenarbeit bewältigen oder mit ökonomischen Auswirkungen umgehen. Auch hier werden teilweise zusätzliche Tätigkeiten berichtet ebenso wie Ängste vor einem Arbeitsplatzverlust [17].

## BGF in Inklusionsbetrieben

Im Allgemeinen stellt der Arbeitsplatz als Setting einen Lebensbereich dar, an dem ein großer Teil der jeweiligen Zeit verbracht wird und ein entsprechend großer Einfluss auf die Gesundheit verzeichnet werden kann [40]. Für Menschen mit Behinderungen ist in der UN-Behindertenrechtskonvention festgelegt, dass sowohl Präventionsangebote als auch gesundheitsförderliche Arbeitsbedingungen bereitgestellt werden sollen [2]. Zum Stand der BGF in Inklusionsbetrieben zeigen erste Ergebnisse von Sommer et al. [31], dass bereits 71,0 % (*n* = 93 Inklusionsbetriebe) die BGF im Bereich der Arbeitsbedingungen oder in der Unternehmenskultur (50,5 %) umsetzen. Weitere verhaltensbezogene Angebote werden von 45,2 % der Inklusionsbetriebe angeboten. Zusätzlich berichten rund 80,0 %, dass sie kein individuelles BGF-Konzept im Vergleich zu bereits bestehenden Angeboten der gesetzlichen Krankenkassen nutzen. Jedoch erläutern andere Inklusionsbetriebe, dass weitere lokale Angebote gefordert werden oder dass die bestehenden Angebote die individuelle Situation des Betriebs bzw. die spezifischen Bedarfe der Beschäftigten nicht ausreichend berücksichtigen [31].

Eine erste Bedarfsanalyse mittels qualitativer Einzel- bzw. Gruppenbefragungen von Beschäftigten, Leitungskräften, Expertinnen und Experten zeigt zudem bestehende Angebote zur BGF sowie Verbesserungsbedarfe und Herausforderungen bei der Umsetzung [18]. In Inklusionsbetrieben werden bereits unterschiedliche Maßnahmen zur BGF in den Bereichen Bewegung (z. B. verschiedene Sportgruppen), Ernährung (z. B. Kochkurse), Entspannung (z. B. Massagen), Kooperationen (z. B. Gesundheitschecks oder Aktionen) sowie Schulungen und Seminare zu verschiedenen Themen umgesetzt. Neben eher allgemein formulierten Verbesserungsbedarfen in den oben genannten Bereichen, etwa zur Nutzung von Belohnungen, Unterstützung bei der Umsetzung einer gesunden Ernährung oder weiteren Angeboten zur Entspannung, werden auch Bedarfe spezifisch für Menschen mit Behinderung am ersten Arbeitsmarkt dargestellt, z. B. Kurse zum Stressmanagement in leicht verständlicher Sprache bzw. Trainer:innen, die selbige nutzen können [18]. In der Literatur wird zudem bemängelt, dass insbesondere Menschen mit komplexeren Behinderungen schlechter von Interventionen erreicht werden, wie etwa Menschen mit Lern-, geistigen oder mehrfachen Behinderungen sowie diejenigen mit Migrationshintergrund [1].

Auch im Bereich der Herausforderungen werden für Inklusionsbetriebe auf der einen Seite bereits aus der Literatur bekannte Aspekte genannt wie etwa eine geringe Teilnahmebereitschaft, mangelnde Struktur oder fehlende ganzheitliche Vorgehensweisen in bestehenden Angeboten [18]. Aktuelle Studien zeigen beispielsweise Teilnahmequoten von < 50 % [26] oder berichten, dass nur 9 % im Anschluss an eine Analysephase verhaltens- und verhältnisbezogene Maßnahmen anbieten [3]. Weitere Herausforderungen bestehen durch eine mangelnde Unterstützung seitens der Geschäftsführung als wesentlicher Akteur bei der Umsetzung von BGF [27, 39]. Für Inklusionsbetriebe und die dort vorherrschende Verpflichtung zur BGF zeigt sich, dass es an (zeitlichen, finanziellen oder personellen) Ressourcen mangelt. So wird berichtet, dass für Leitungskräfte in Inklusionsbetrieben (etwa im Vergleich zu WfbM) das betriebliche Kerngeschäft im Fokus steht; insbesondere vor dem Hintergrund des bestehenden ökonomischen Drucks am ersten Arbeitsmarkt [18].

Auf der anderen Seite wird deutlich, dass eine zusätzliche Förderung durch die Integrationsämter und den Mitteln der Förderrichtlinie *AlleImBetrieb* bereitgestellt wurde, die den Ausbau der BGF oder der beruflichen Weiterbildung vorsieht [6]. Sommer et al. beschreiben jedoch, dass diese Mittel bisher nur wenig abgerufen werden, etwa aufgrund von fehlenden Anträgen, niedriger Nachfrage, teilweise unklarer Vorgaben oder fehlender Leitfäden zur Förderungsantragstellung. Zudem können Anträge nur für Beschäftigte der Zielgruppe genutzt werden oder es werden bereits andere Landesförderprogramme bereitgestellt [31]. Des Weiteren sind bestehende Weiterbildungen zumeist nicht an die Rahmenbedingungen in Inklusionsbetrieben angepasst (sondern eher auf WfbM ausgelegt), es fehlt an Angeboten in leicht verständlicher Sprache und gehörlose Beschäftigte werden häufig nicht ausreichend berücksichtigt. Andere Herausforderungen entstehen in Bezug auf die verschiedenen Bedürfnisse und Aufnahmefähigkeiten der Beschäftigten, die eine Standardisierung von Angeboten erschweren [18]. Letztlich beeinträchtigen auch verschiedene Arbeitszeitmodelle der Beschäftigten, welche etwa aufgrund von psychischen Erkrankungen bestehen, die Umsetzung von Angeboten der BGF. Andere Autorinnen und Autoren untermauern diese Ergebnisse und weisen darauf hin, dass Überstunden, Arbeiten in Teilzeit, unregelmäßige Arbeitszeiten, begrenzte Möglichkeiten, den Arbeitsplatz zu verlassen, oder mehrere Unternehmensstandorte die Umsetzung von Maßnahmen der BGF hemmen [23, 27, 39].

Eine erste Evaluationsstudie aus dem Modellvorhaben ‚Betriebliche Gesundheitsförderung in Inklusionsbetrieben‘ (BeGIn) nach §§ 215 ff. SGB IX adressiert die spezifischen Bedarfe von Inklusionsbetrieben im Bereich der BGF [11]. Die Konzeption verhaltenspräventiver BGF-Angebote sowohl für Beschäftigte mit Behinderungen in leicht verständlicher Sprache als auch für deren Leitungskräfte ist dabei auf die bestehenden Anforderungen und Ressourcen in Inklusionsbetrieben abgestimmt. Für Beschäftigte mit Behinderungen wurden drei Seminarkonzepte zur Selbstfürsorge im Arbeitsalltag, gesunden Teamarbeit und Kommunikation und Konfliktlösung entwickelt – für Leitungskräfte ein Seminarkonzept zur gesunden Führung, in welchem u. a. der Umgang mit Konflikten zwischen wirtschaftlichen und sozialen Anforderungen, eigenen Abgrenzungsstrategien sowie wertschätzende Kommunikation thematisiert wird. Die Evaluationsergebnisse zeigen, dass alle durchgeführten BGF-Maßnahmen von den Beschäftigten mit Behinderungen sowie der Leitungskräfte mehrheitlich positiv bewertet werden. Dabei werden insbesondere die auf Inklusionsbetriebe angepassten Seminarinhalte und -organisation von den Teilnehmenden wertgeschätzt. Insgesamt verdeutlicht diese Evaluationsstudie, dass Seminare zur Förderung der psychischen Gesundheit eine geeignete Möglichkeit darstellen, verhaltenspräventive BGF-Maßnahmen umzusetzen [11].

## Ausblick

In Abgrenzung zu anderen Beschäftigungsmöglichkeiten sowohl in WfbM als auch in anderen Betrieben auf dem ersten Arbeitsmarkt zeigen sich Gemeinsamkeiten aber auch Unterschiede hinsichtlich der Arbeitsanforderungen und Ressourcen von Beschäftigten mit Behinderungen und ihren Leitungskräften in Inklusionsbetrieben [10, 16, 19]. Zusammenfassend wird deutlich, dass Inklusionsbetriebe den Vorteil bieten, die Arbeitsbedingungen flexibel an die individuellen Voraussetzungen von Beschäftigten anzupassen. Beschäftigte mit Behinderungen beschreiben jedoch ebenfalls Arbeitsanforderungen, beispielsweise im Bereich der Arbeitsorganisation und -umgebung. Spezifisch für das Setting von Inklusionsbetrieben werden ebenfalls vorteilhafte Bedingungen sichtbar: Erfüllende Arbeitsaufgaben, die Zugehörigkeit zu einem produktiven Team, soziale Unterstützung, Arbeitsplatzsicherheit und verlässliche Bezahlungen sowie Entwicklungsmöglichkeiten kennzeichnen die Ressourcen für Beschäftigte [10]. Ebenfalls kann eine spezifische Anforderungssituation für Leitungskräfte in Inklusionsbetrieben dargestellt werden. Zwar begegnen Leitungskräfte in Inklusionsbetrieben genauso wie in anderen Betrieben auf dem ersten Arbeitsmarkt wirtschaftlichen Herausforderungen, jedoch werden Herausforderungen in der Balance zwischen sozialen und wirtschaftlichen Anforderungen durch die Leitung von Beschäftigten mit und ohne Behinderung deutlich [16].

**Abb. 1:**
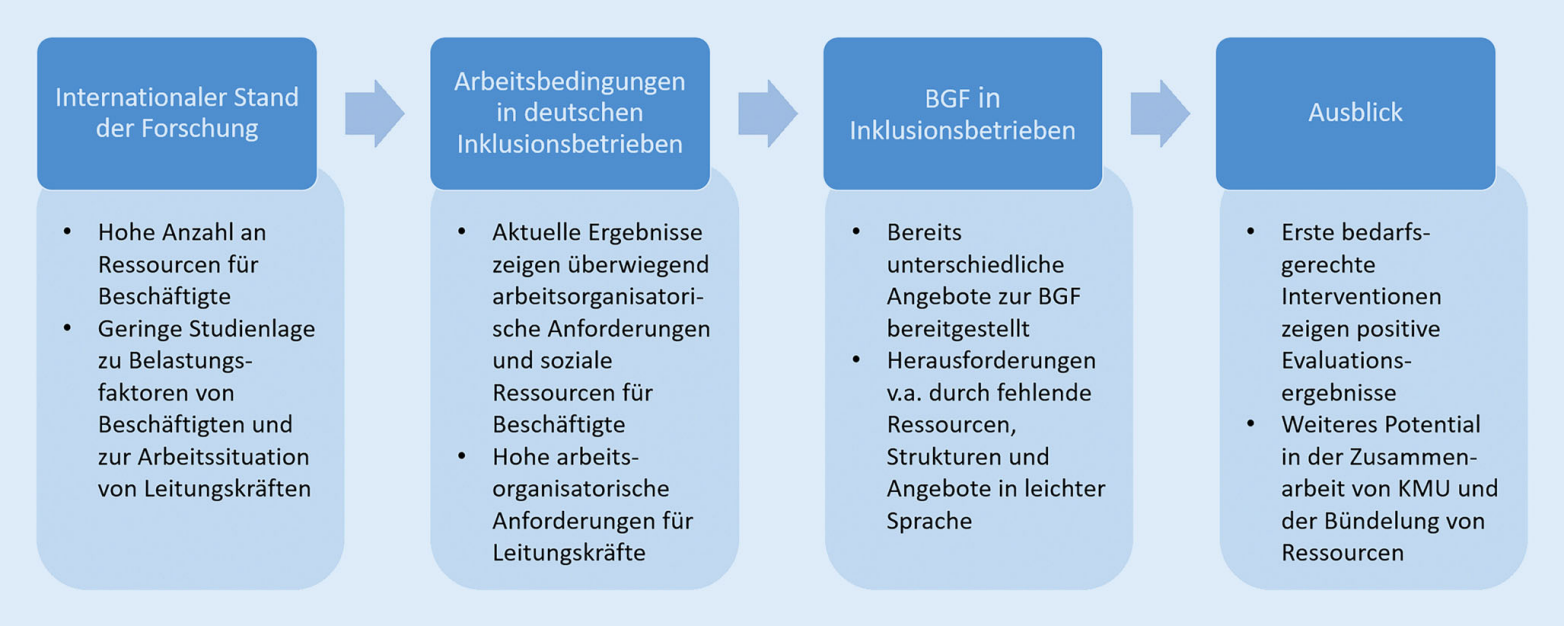
Übersicht zum aktuellen Stand der Forschung zu den Arbeitsbedingungen und zur betrieblichen Gesundheitsförderung (BGF) in Inklusionsbetrieben

Die dargestellte erste Bedarfsanalyse zur BGF im Modellvorhaben BeGIn untermauert weitere Handlungsfelder im Bereich der BGF in Inklusionsbetrieben [18], um den derzeitigen Herausforderungen wie fehlende Ressourcen, Strukturen oder angepasste BGF-Maßnahmen (z. B. hinsichtlich der Arbeitsorganisation, des zeitlichen Umfangs, der Anpassung an individuelle Bedarfe) zu begegnen. Besonders den Geschäftsführungen kommt bei der Umsetzung der BGF eine zentrale Verantwortlichkeit zu [28]. Aufgrund der oftmals fehlenden Ressourcen insbesondere in klein- und mittelständischen Unternehmen und der Priorisierung des Tagesgeschäfts können zur Entlastung der Betriebe Kooperationen zwischen den Betrieben und weiteren Akteuren angeregt werden, welche beispielsweise Unterstützung durch Koordinatorinnen und Koordinatoren, BGM-Expertinnen und -Experten und Sozialversicherungsträger umfassen. Insbesondere durch die Vergabe von Koordinationsaufgaben könnte sowohl die Organisation von gemeinsamen Treffen erleichtert werden als auch die Umsetzung von Maßnahmen zur BGF [28]. In diesem Zusammenhang erweist sich besonders das spezifische, auf die Arbeits- und Gesundheitssituation von Beschäftigten mit Behinderungen und ihren Leitungskräften in Inklusionsbetrieben angepasste Angebot als relevant (z. B. Vermittlung in leicht verständlicher Sprache). Weitere Ansätze zur Förderung der BGF in Inklusionsbetrieben können durch Datenbanken von Trainer:innen entstehen, die leicht verständliche Sprache verwenden können [18]. Abb. 1 zeigt eine Übersicht zum aktuellen Stand der Forschung zu den Arbeitsbedingungen und zur BGF in Inklusionsbetrieben.

## Fazit für die Praxis

- Im Vergleich zu anderen Beschäftigungsmöglichkeiten werden in Inklusionsbetrieben verschiedene Arbeitsanforderungen und Ressourcen für Beschäftigte mit Behinderungen und deren Leitungskräften dargestellt.
- Derzeit besteht noch Forschungsbedarf und Verbesserungspotenzial in der Praxis zur betrieblichen Gesundheitsförderung (BGF) in Inklusionsbetrieben. Herausforderungen bestehen insbesondere hinsichtlich fehlender Ressourcen, Strukturen und angepasster Angebote, z. B. in leicht verständlicher Sprache.
- Im Bereich der BGF besteht für Inklusionsbetriebe das Potenzial, ihre Ressourcen unter Einbezug der Unterstützung durch Sozialversicherungsträger in Netzwerken zu bündeln. Dazu wird beispielsweise angeregt, Koordinationsaufgaben zu vergeben, um gemeinsame Treffen zu planen und Maßnahmen zur BGF umzusetzen.
